# Effect of Addition of Tryptophan on Aggregation of Apo-α-Lactalbumin Induced by UV-Light

**DOI:** 10.3390/foods10071577

**Published:** 2021-07-07

**Authors:** Zichen Zhao, Renjie Li, Mahesha M. Poojary, Søren B. Nielsen, Marianne N. Lund

**Affiliations:** 1 Department of Food Science, Faculty of Science, University of Copenhagen, Rolighedsvej 26, 1958 Frederiksberg, Denmark; zichen@food.ku.dk (Z.Z.); renjie@food.ku.dk (R.L.); mahesha@food.ku.dk (M.M.P.); 2 Arla Foods Ingredients Group P/S—Innovation R&D, Discover Department, Sønderupvej 26, 6920 Videbæk, Denmark; sobni@arlafoods.com; 3 Department of Biomedical Sciences, Faculty of Health and Medicine, University of Copenhagen, Blegdamsvej 3, 2200 Copenhagen, Denmark

**Keywords:** UV illumination, tryptophan, aggregation, photooxidation, whey protein

## Abstract

UV-B illumination facilitates aggregation of alpha-lactalbumin (α-LA) by intramolecular disulfide bond cleavage followed by intermolecular thiol-disulfide exchange reactions. However, long term exposure to UV-B illumination may induce undesired oxidative modifications of amino acid residues in the protein. The purpose of this study was to examine the effect of UV-induced aggregation of apo-α-LA (a calcium-depleted form of α-LA) under aerobic and anaerobic conditions and by addition of tryptophan (Trp) as a photosensitizer. The addition of Trp to apo-α-LA illuminated under anaerobic conditions facilitated the highest level of free thiol release and disulfide-mediated aggregation as compared to without addition of Trp under both anaerobic and aerobic conditions. Addition of Trp under aerobic condition resulted in the lowest level of free thiols and disulfide-mediated aggregation and the aerobic conditions caused oxidation of the free Trp with formation of kynurenine and 5-hydroxy-Trp. Minor levels of the Trp oxidation product, 3-hydroxy-kynurenine (2% converted from Trp), was formed in apo-α-LA with added Trp under both aerobic and anaerobic conditions after UV-B treatment.

## 1. Introduction

Whey proteins are the by-products of cheese and casein manufacture. They are widely used as food ingredients owing to their high nutritional value and versatile functional properties such as gelation, foam stabilization, and emulsification [1,2,3,4]. Alpha-lactalbumin (α-LA) is one of the major proteins present in whey together with beta-lactoglobulin (β-LG) [4]. Prior to application, whey proteins may be thermally treated to form protein aggregates to increase their stability in the final food or beverage product. However, the thermal treatment may induce adverse effects such as the formation of off-flavors [5,6]. UV treatment has, therefore, been examined as an alternative tool to produce whey protein aggregates [7].

As opposed to β-LG, native α-LA does not contain any free thiol group, since all cysteine (Cys) residues are present as disulfides [8]. α-LA is, therefore, less prone to undergo thermal aggregation via thiol-disulfide exchange reactions compared to β-LG. It is an excellent model protein to study the influence of UV light without interference from thiol-disulfide exchange reactions initiated from free Cys residues. By UV-B illumination, intramolecular disulfide bonds in α-LA are cleaved and free thiol groups released, which may then undergo thiol-disulfide exchange reactions and facilitate aggregation through the formation of intermolecular disulfide bonds [7]. It has been reported that UV-B light can induce aggregation of apo-α-LA (a calcium-depleted form of α-LA), with 98% of the monomer of apo-α-LA being converted to aggregates after UV illumination for 24 h [7]. However, significant levels of oxidation were observed on several of the amino acid residues (Cys, methionine (Met), tryptophan (Trp), tyrosine (Tyr), histidine (His), and phenylalanine (Phe)) of the generated α-LA aggregates, and it is therefore of interest to optimize the UV illumination to avoid oxidative damage of proteins.

The exact mechanism for the UV-induced disulfide bond cleavage in α-LA is not known, but several mechanisms are likely; the disulfide bonds may absorb UV light directly and undergo a homolytic cleavage [9,10], and/or Trp and Tyr residues in α-LA may absorb the light in the near-UV region of the spectrum [11,12,13], and hereby act as a photosensitizer [14]. This leads to excited states Trp and Tyr and release of solvated electrons [14,15], which have been shown to cleave the disulfide bonds in α-LA and release free thiols [11,13]. In a previous study, we have shown that it is possible to achieve an efficient cleavage of disulfide bonds and release of free thiols (close to 50%) in an aqueous solution of cystine and Trp under anaerobic conditions after UV light treatment [16]. The apparent quantum yields of free thiol formation obtained in the study with free amino acids showed that the release of free thiols was more efficient under anaerobic conditions. In addition, the cleavage of disulfide bonds was more efficient when cystine was irradiated in the presence of Trp (1:1 molar ratio) compared to Tyr (1:1 molar ratio) or cystine alone [16]. Nevertheless, increasing the molar concentration of the Trp to 2:1 ratio with respect to cystine resulted in lower quantum yield for the release of free thiols and higher quantum yields for degradation of Trp, which indicated enhanced oxidation of Trp [16]. The aim of the present study was therefore to investigate the effect of addition of free Trp on UV-B-induced aggregation of apo-α-LA under both aerobic and anaerobic conditions. The corresponding oxidative modification of aromatic acid residues in apo-α-LA was examined.

## 2. Materials and Methods

### 2.1. Chemicals and Reagents

Bovine α-LA containing 0.1 mole of Ca^2+^ per mole of α-LA (product number L6010), L-Trp, 4-(2-hydroxyethyl)-1-piperazineethanesulfonic acid (HEPES) (≥99.5%), 4,4′-dithiodipyridine (4-DPS) (98%), l-glutathione (GSH) (≥98%), 1,4-dithiothreitol (DTT), methanesulfonic acid (MSA), tryptamine (≥97%), boric acid, 5-hydroxy-l-Trp (5-OH-Trp), l-kynurenine (Kyn), *N*-formylkynurenine (NFK), 3-hydroxy-dl-kynurenine (3-OH-Kyn), 3,4-dihydroxy-l-phenylalanine (DOPA), dl-*o*-tyrosine (*o*-Tyr), dl-*m*-tyrosine (*m*-Tyr), sodium tetraborate (99%), methanol (≥99.9%), 5-methyl-dl-tryptophan (≥97%), sodium perchlorate monohydrate (ACS reagent), phosphoric acid solution (85%), and hydrochloric acid (37%) were obtained from Sigma-Aldrich (St. Louis, MO, USA). Calcium chloride, sodium dihydrogen phosphate, sodium hydroxide, sodium chloride, ethylenediaminetetraacetic acid disodium salt dihydrate (EDTA), urea, and acetonitrile (≥99.9%) were obtained from Merck (Darmstadt, Germany). LL-Di-tyrosine (di-Tyr) dihydrochloride was purchased from Toronto Research Chemical (Ontario, Canada). Sodium dodecyl sulfate (ultra-pure) was purchased from MP biomedicals (Illkirch, France). All other reagents were of analytical grade. All aqueous solutions were made from purified water obtained from a Millipore Milli-Q purification system (Millipore Corporation, Billerica, MA, USA).

### 2.2. Sample Preparation and Illumination

Stock solutions of apo-α-LA (0.705 mM) were prepared with 0.5 mM EDTA, with or without the addition of 2.8 mM Trp in 50 mM HEPES buffer and adjusted to pH 7.0. This resulted in a 2:1 ratio of Trp to disulfide bonds in the sample with added Trp since α-LA itself contains 4 Trp residues and 4 disulfide bonds. Minimum three independent stock solutions were prepared for each sample under aerobic and anaerobic conditions, as previously described [7]. Anaerobic conditions were achieved by preparing all sample solutions in an anaerobic chamber (Coy Laboratory Products, Grass Lake, MI, USA) by using degassed HEPES buffer (bubbled with nitrogen for 4½ h before use). The oxygen concentration in these solutions was 6 ± 1 µM (measured by an HQ30D portable dissolved oxygen meter (Hach, Loveland, CO, USA). The protein concentration of each stock solution was determined by a NanoDrop 1000 spectrophotometer (Thermo Fisher Scientific, Wilmington, Delaware, DE, USA) by measuring absorbance at 280 nm and using an extinction coefficient of ε^1%^ = 20.1 [7]. Each α-LA (3 mL) was transferred into transparent quartz cuvettes (Hellma Analytics, Müllheim, Germany), and three cuvettes were placed in the bottom of a Rayonet Photochemical Reactor equipped with 3000 Å lamps (The Southern New England Ultraviolet CO, Branford, CT, USA) and a magnetic stirrer as described previously [7]. Samples were illuminated with constant stirring at 1000 rpm for up to 24 h with aliquots of 200 µL taken after 2, 4, 6, and 24 h. Samples without illumination at 0 h were regarded as control samples. Immediately after illumination, samples were snap-frozen in liquid nitrogen and stored at −80 °C until analysis. All samples were illuminated in independent triplicates.

### 2.3. Characterization of Protein Aggregates by Gel Electrophoresis

Loading samples of illuminated apo-α-LA with or without the addition of Trp under aerobic and anaerobic conditions were prepared by the addition of NuPAGE^®^ LDS sample buffer (4×, Thermo Fisher Scientific, Carlsbad, CA, USA). DTT or MilliQ water was added to obtain reduced or non-reduced samples, respectively [17]. Unstained protein molecular marker (mass range, 14.4–116 kDa or 10–200 kDa Thermo Fisher Scientific, Rockford, IL, USA) was loaded to each gel. Sodium dodecyl sulfate–polyacrylamide gel electrophoresis (SDS-PAGE) was performed using precast 15-well NuPAGE^TM^ 4–12% Bis-Tris gels from Thermo Fisher Scientific (Carlsbad, CA, USA), as described by Zhao et al. [7].

### 2.4. Determination of the Molecular Mass Distribution of Protein Aggregates by Size Exclusion Chromatography (SEC)

The yield of protein aggregates formed by illumination was quantified by SEC by separating monomers and aggregates on a bioZen SEC-3, 300 × 4.6 mm column (Phenomenex, Torrance, CA, USA). The mobile phase consisted of sodium phosphate buffer (pH 6.8, 100 mM). The yield of total aggregates formed by both non-covalent and covalent binding was determined directly from illuminated and non-illuminated apo-α-LA samples, while aggregates formed by covalent cross-links were determined from samples added 75 µL of 5% SDS into 75 µL of illuminated and non-illuminated samples. In addition, the yield of aggregates formed by only non-reducible cross-links were determined by adding 75 µL of 5% SDS mixed with 1 M DTT to the same volume of samples. Samples with added SDS (with or without DTT) were heated in a heating block Provocell (Esco, Gauteng, South Africa) for 5 min at 90 °C prior to injection. All samples were filtered through a membrane filter (0.45 µm, Millipore, San Jose, CA, USA) before injecting 0.5 mL of each sample onto the column. Analytes were eluted with the mobile phase at a flow rate of 0.1 mL/min for 60 min at 18–22 °C and detected at 280 nm. The content of aggregates (retention time < 26 min), monomers (retention time 26–34 min), and fragments (molecular weight less than monomers) (retention time 34–43 min) in the samples was calculated based on an external calibration curve ranging from 0 to 20 mg/mL of α-LA. The fractions (in %) of aggregates, or monomers, or fragments for each sample were calculated by dividing the area under the curve for each fraction by the total area under the curve of all peaks in the chromatogram.

### 2.5. Quantification of Free Thiols by 4,4′-Dithiodipyridine (4-DPS) Derivatization

The concentrations of free thiols released after illumination of apo-α-LA with or without the addition of Trp and prepared under aerobic and anaerobic conditions were determined by reaction with 4 mM 4-DPS as described previously [7]. The illuminated samples were diluted 10 times in phosphate buffer containing urea (100 mM NaH_2_PO_4_, 0.2 mM EDTA, 5 M urea, pH 7.0). The absorbance of the samples derivatized with 4-DPS was determined at 324 nm with a SpectraMax i3x Multi-Mode Microplate Reader at 25 °C (Molecular Devices, San Jose, CA, USA). The amount of mol thiol/mol protein in the samples was calculated based on an external calibration curve ranging from 0 to 48 µM of glutathione (GSH). Three different independent replicates were prepared, each of which contained three technical replicates. The results were presented as mean ± standard deviation (mol thiol/mol protein).

### 2.6. Quantification of Oxidation Products from Trp, Tyr, and Phe

The oxidation products of Trp, Tyr, and Phe residues in illuminated α-LA samples were analyzed as described in Zainudin et al. [18]. This method included the detection of 3-OH-Kyn, 5-OH-Trp, Kyn, and NFK (oxidation products of Trp), DOPA, and di-Tyr (oxidation products of Tyr), and *m*-Tyr and *o*-Tyr (oxidation products of Phe). Free Trp and proteins in the samples were separated by a VIVA SPIN 500 spin filter (10,000 molecular weight cut-off, polyether sulfone (PES) membrane), (Sartorius, Stedim Lab Ltd., Stonehouse, UK) prior to analysis. The supernatant containing free Trp was preserved, while the solution containing protein was eluted with 2 mL of 50 mM HEPES buffer and then centrifuged at 12,100× *g* (Eppendorf Nordic A/S, Hørsholm, Denmark) for 40 min four times. The resultant protein fraction was hydrolyzed by 300 µL of 4 M MSA containing 0.2% (*w*/*v*) tryptamine in 2 mL microwave reaction vials (Biotage, Uppsala, Sweden) as described previously [7]. The hydrolysates were neutralized with 4 M sodium hydroxide. The neutralized protein hydrolysates and the fraction containing free Trp obtained from centrifugal filtration were independently spiked with 100 μL of 50 μg/μL internal standard, 5-methyl-dl-Trp, and filtered through a 0.2 µm filter before injection (5 µL) onto a UHPLC system equipped with a Syncronis aQ C18 column (2.1 ID × 100 mm length, 1.7 µm particles) (Thermo Fisher Scientific, Torrance, CA, USA), as described by Zhao et al. [16].

### 2.7. Statistical Analysis

Results were shown as means ± standard deviation (SD) of three independent replicates. Statistical analysis was carried out by using one-way ANOVA with a Tukey post-hoc test in IBM SPSS Statistics 23 (IBM, New York, NY, USA), with *p* < 0.05 considered as statistically significant.

## 3. Results and Discussion

### 3.1. UV-B-Induced Aggregation of Apo-α-LA in the Absence and Presence of Free Trp and Oxygen

**Figure 1:**
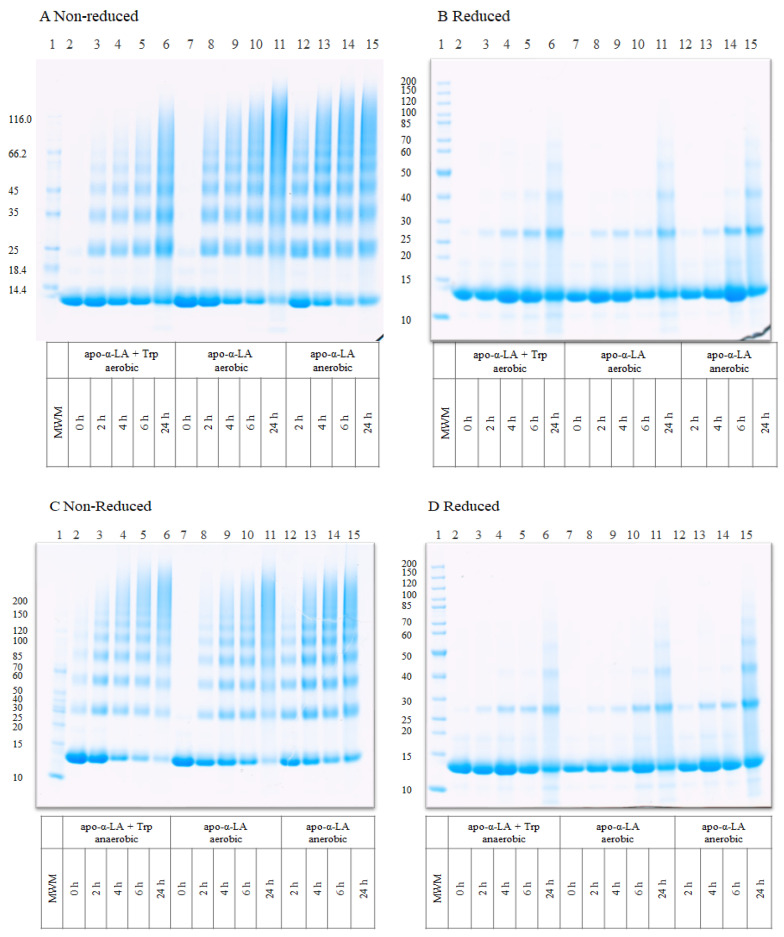
SDS-PAGE of apo-α-LA with or without the addition of 2.8 mM Trp prepared under aerobic and anaerobic conditions in 50 mM HEPES buffer, pH 7.0 after UV-B illumination for 0, 2, 4, 6, and 24 h. (**A**) Non-reduced samples of apo-α-LA with added free Trp under aerobic conditions and apo-α-LA under aerobic and anaerobic conditions. (**B**) Reduced apo-α-LA samples as described in A. (**C**) Non-reduced samples of apo-α-LA with added free Trp under anaerobic conditions and apo-α-LA under aerobic and anaerobic conditions. (**D**) Reduced apo-α-LA samples as described in C. Reduced samples were added DTT, and all gels were stained with Coomassie brilliant blue. Molecular weight markers (MWM) were included on each gel. Representative gels of three independent experiments are shown.

SDS-PAGE was used to visualize apo-α-LA aggregates formed by UV-B light exposure (Figure 1). Increased intensities of bands with oligomers (18–66 kDa) or aggregates (>66 kDa) and a simultaneous decrease in the intensity of monomeric bands (14.1 kDa) was observed with increasing time of UV treatment under both aerobic and anaerobic conditions and in the presence and absence of Trp (Figure 1A,C). Increased levels of oligomers and aggregates of apo-α-LA were obtained after UV treatment from 2 to 24 h in the absence of Trp (lanes 8–11) than in the presence of Trp (lanes 3–6) under aerobic conditions (Figure 1A).

This was also evident from the evaluation of the monomer band intensity, which indicated a greater loss of α-LA monomer after 24 h of illumination in the absence of Trp (comparison between lanes 6 and 11; Figure 1A). Under anaerobic conditions, the presence of Trp resulted in a greater loss of monomer band (lanes 3–6) as compared to in the absence of Trp (lanes 12–15) (Figure 1C). These results suggest that the addition of free Trp did not promote aggregation of apo-α-LA by UV treatment under aerobic conditions, but potentially did have a promoting effect under anaerobic conditions. Nevertheless, there were no clear differences in the formation of oligomers and aggregates with and without the addition of free Trp under anaerobic conditions. The extent of oligomers and aggregates of apo-α-LA formed under aerobic and anaerobic conditions in the absence of Trp was quite similar (comparison between lanes 7–11 and 12–15 in both Figure 1A,C), but a greater loss of monomer band was observed under aerobic conditions. This observation suggested that UV illumination under aerobic conditions (without addition of Trp) led to a greater extent of aggregation. When the samples were reduced by DTT (Figure 1B,D), it was clearly observed that most of the aggregate bands were lower in their intensities for all samples, indicating that the covalent cross-links formed in the aggregates were mostly reducible (primarily disulfide bonds), in agreement with our previous study [7]. The prominent dimer band between 25–30 kDa and a less intense trimer band at ~40 kDa, especially after 24 h, is likely caused by formation of non-reducible di-Tyr, di-Trp, and Trp-Tyr cross-links as observed previously [7]. Fragmentation was also observed as indicated on the reduced gel as faint bands below the monomer band and was more apparent than in non-reduced samples indicating that fragments were also cross-linked by disulfide bonds. The observed fragmentation may be induced by peptide bond cleavage mediated by electrons or radical formation after UV treatment, as previously described [19,20].

**Figure 2:**
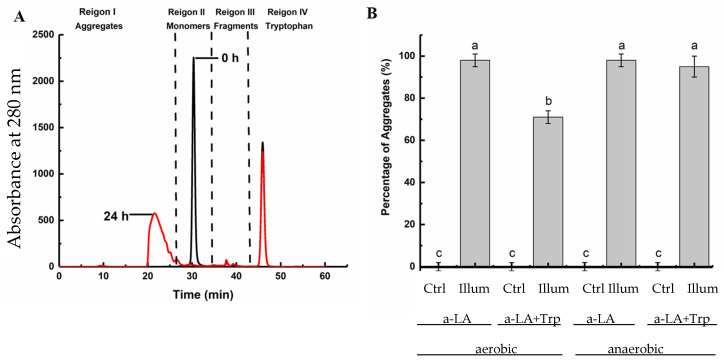
(**A**) Chromatogram of apo-α-LA in the presence of 2.8 mM Trp under anaerobic conditions after 0 h (black) and 24 h illumination (red). (**B**) Yield of total aggregates formed in illuminated apo-α-LA in the absence or presence of 2.8 mM Trp under aerobic and anaerobic conditions. Results are presented as mean values obtained from three independent experiments with standard deviations shown by error bars. Means with different letters are significantly different.

Due to difficulties in assessing the extent of aggregation by SDS-PAGE analysis, the samples were also analyzed by SEC firstly without addition of SDS. Non-covalent bonds are suppressed by SDS during the electrophoresis and are therefore not visualized on the SDS-PAGE gels. Three groups of peaks were observed in the chromatograms: peaks with retention time < 26 min were regarded as aggregates, the sharp peak at 26–34 min was the α-LA monomer, peaks eluting after 34–43 min were regarded as fragments, and added Trp eluted after 43 min. A representative chromatogram is shown in Figure 2A. No aggregates were observed in all the control samples, but after 24 h of UV-B treatment, significant levels of aggregation were observed in all the samples (Figure 2B), which was consistent with the results obtained from SDS-PAGE analysis (Figure 1). In agreement with our previous study [7], 98% of the monomers were converted to aggregates in apo-α-LA samples without added Trp after UV treatment under aerobic conditions, and the same extent of aggregation was observed under anaerobic conditions.

When Trp was added to apo-α-LA, 95% of the monomers were converted to aggregates after UV treatment under anaerobic conditions, but this result was not significantly different (*p* > 0.05) from samples without added Trp. As opposed to the results of the SDS-PAGE gels, monomers were only observed in the sample of apo-α-LA with added Trp illuminated under aerobic conditions. The lack of monomers in the other illuminated samples is likely due to the absence of SDS in the SEC analysis of results presented in Figure 2, which allows inclusion of aggregates formed by non-covalent bonds in the SEC analysis.

Under aerobic conditions, only 71% of the monomers were converted to aggregates in apo-α-LA with added Trp, indicating that the combination of free Trp and oxygen disrupted the aggregate formation (Figure 2B). This observation was in agreement with the observed degree of monomers in the SEC analysis (26%) and the SDS-PAGE results where more monomeric protein remained in the gels after UV illumination with added Trp under aerobic conditions as compared to anaerobic conditions. A possible explanation of this could be that the released free thiol groups were further oxidized to sulfinic or sulfonic acids by the increased oxidation likely to occur with the presence of both free Trp and oxygen, and therefore, less disulfide bond cross-links were formed, resulting in a lower degree of aggregate formation. No significant differences in the formation of fragments could be observed between the different samples as the fragment levels were very low.

### 3.2. Formation of Reducible Covalent Cross-Links and Release of Free Thiols during UV-B Illumination

**Figure 3:**
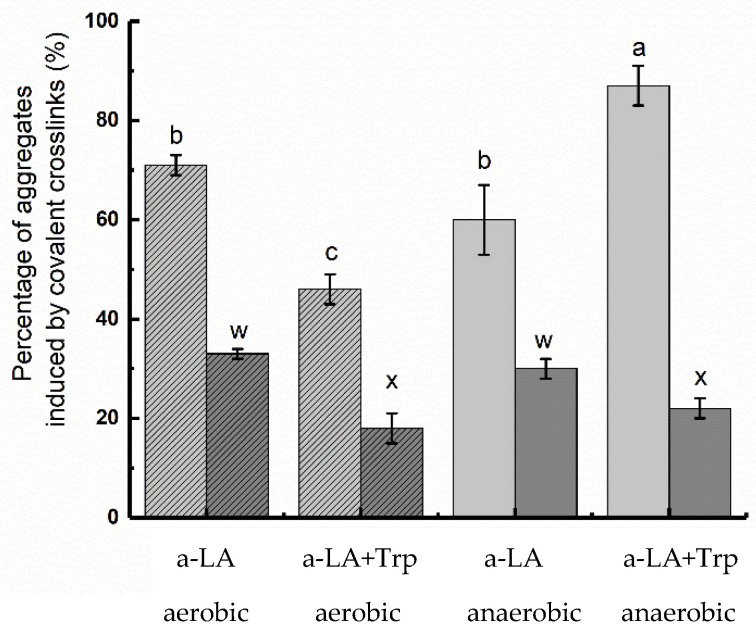
Covalent cross-links formed in apo-α-LA in the absence or presence of 2.8 mM Trp under aerobic and anaerobic conditions obtained after the addition of 5% SDS to disrupt non-covalent interactions. Non-reduced samples are shown in light grey bars, while samples reduced by DTT are shown in dark grey bars. Results are presented as mean values obtained from three independent experiments with standard deviations shown by error bars. Bars with different letters are significantly different.

The yield of aggregates formed by only covalent cross-links (reducible and non-reducible) was determined by SEC analysis after the illuminated samples had been added SDS and DTT in order to disrupt non-covalent and reducible covalent cross-links, respectively, in the aggregates (Figure 3).

All samples had higher levels of aggregates in non-reduced samples as compared to reduced samples, which supported the SDS-PAGE results showing the formation of primarily reducible cross-links in the aggregates. For samples without added Trp (both non-reduced and reduced), there was no significant difference (*p* > 0.05) in the percentage of covalently linked aggregates formed between apo-α-LA illuminated under aerobic and anaerobic conditions. Interestingly, addition of Trp and illumination under anaerobic conditions significantly increased the level of aggregates formed by covalent cross-links (to 87%) compared to all other treatments. On the other hand, addition of Trp under aerobic conditions resulted in the lowest percentage of covalent cross-links. The additional contribution to covalent cross-links observed for samples added Trp under anaerobic conditions was found to be caused by formation of reducible cross-links (i.e., primarily disulfides) as the difference between reduced and non-reduced samples was the largest compared to the other samples. A possible explanation of this observation is that addition of Trp resulted in an increase in the concentration of excited state Trp and/or solvated electrons leading to a higher degree of disulfide bond cleavage in apo-α-LA and thus the possibility to mediate thiol-disulfide exchange reactions and covalent disulfide-mediated aggregation. In the samples added Trp under aerobic conditions, it is possible that oxygen either quenched excited state Trp and/or gain free electrons, which would decrease the level of disulfide cleavage, or that the free thiols released from disulfide cleavage would be further oxidized [7,16]. The percentage of non-reducible covalent cross-links was not significantly different between samples added Trp illumination under aerobic and anaerobic conditions indicating that the effect of Trp and oxygen only affected disulfide-mediated aggregation and not other non-reducible cross-links.

**Figure 4:**
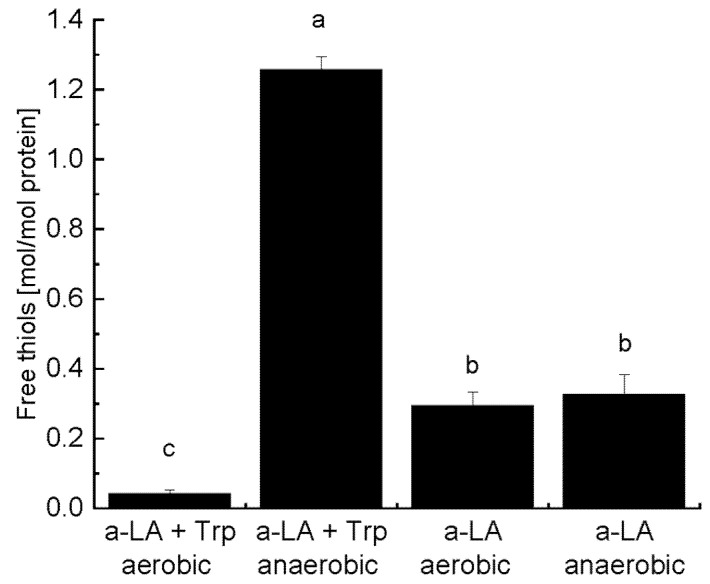
Free thiols (mol/mol apo-α-LA) released in apo-α-LA in the presence or absence of 2.8 mM Trp under aerobic and anaerobic conditions after 24 h of illumination. Mean values ± SD (standard deviation) are shown. Means with different letters are significantly different (one-way ANOVA with a Tukey posthoc test, *p* < 0.05).

The concentration of free thiols after illumination was also evaluated (Figure 4).

No free thiols could be detected in any of the non-illuminated control samples, which was expected since α-LA does not contain any free Cys residues (data not shown). The level of released thiols in apo-α-LA with added Trp increased to 1.25 ± 0.04 mol/mol apo-α-LA) after 24 h of illumination under anaerobic conditions, which was the highest value among the analyzed samples. The levels of released free thiols in samples without addition of Trp were similar for anaerobic and aerobic conditions (~0.30 mol/mol apo-α-LA), while addition of Trp under aerobic conditions resulted in the lowest level of free thiol release. The levels of free thiols released are consistent with the observed degree of formation of disulfide-mediated cross-links (shown in Figure 3) supporting the previous explanation that addition of Trp under anaerobic conditions facilitates disulfide bond cleavage, release of free thiols [7,21], and disulfide-mediated aggregation, while aerobic conditions cause further oxidation of released thiols [22,23], or that oxygen competes with disulfide bonds for quenching of the excited state of Trp (^3^Trp) or Tyr (^3^Tyr) thereby leading to a lower degree of disulfide bond cleavage and release of free thiols [14,16]. A previous study has also confirmed that the photoexcitation of Trp residues in apo-a-LA induce disulfide bond cleavage and release of thiol groups [13]. UV-irradiation of a 1% whey protein isolate solution has shown to increase the concentration of total thiols (possibly due to disulfide bond cleavage by a similar mechanism as in the present study) and surface-active thiols indicating increased protein unfolding [24].

### 3.3. Formation of Oxidation Products from Aromatic Amino Acids after UV-B Light Treatment

In order to assess the extent of oxidation after illumination of apo-α-LA samples, oxidation products of aromatic amino acid residues were quantified by an established UHPLC method. Since the free Trp added to apo-α-LA could also contribute to the overall degree of oxidation, free Trp was separated from α-LA by spin filtration, and both fractions were analyzed by this method. The fraction containing free Trp was analyzed directly by UHPLC, while the fraction containing α-LA was subjected to acidic hydrolysis prior to UHPLC analysis. No detectable levels of oxidation products were found in the non-illuminated control samples, and no detectable levels of Phe and Tyr oxidation products were found in any of the samples. Oxidative modification of Trp residues in illuminated apo-α-LA was found, but only the oxidation product 3-OH-Kyn was detected after illumination for 24 h under both aerobic and anaerobic conditions (Table 1).

The concentration of 3-OH-Kyn formed in illuminated apo-α-LA was equivalent to the conversion of 2% of the Trp residues in α-LA. In the separated fractions of free Trp, Kyn (58 ± 2 µM), 5-OH-Trp (11 ± 6 µM), and tryptamine (2 ± 0 µM) were formed under aerobic conditions, while only tryptamine (3 ± 1 µM) was formed under anaerobic conditions. The formation of tryptamine was consistent with our previous findings in an aqueous solution of Trp and cystine illuminated under both aerobic and anaerobic conditions [16]. Tryptamine formation occurs by a decarboxylation of Trp via photoionization and is more favorable in the absence of oxygen, while formation of Kyn takes place in the presence of oxygen [25,26]. A previous study has reported the formation of Kyn and NFK after riboflavin-induced Photooxidation of α-LA [27]. No 3-OH-Kyn was detected in the free Trp fraction indicating that the oxidation pathway or stability of oxidation products was different when they originate from proteins and free amino acids. It is likely that the structure of apo-a-LA affects the oxidative reactions occurring in the protein.

## 4. Conclusions

The effect of adding Trp on UV-B-induced aggregation of apo-α-LA was highly dependent on the presence of oxygen; the percentage of aggregates converted from apo-α-LA monomers with added Trp was higher than without added Trp under anaerobic conditions, while addition of Trp under aerobic conditions decreased the formation of aggregates. Aerobic conditions also induced oxidation of the added Trp, while the same degree of oxidation was found on apo-α-LA under aerobic and anaerobic conditions. Aggregates were mainly formed by covalent cross-links, primarily by reducible disulfide bonds, and to a lower extent by non-covalent interactions. Overall, this study shows that the addition of Trp to apo-α-LA only improves the formation of covalently bound aggregates under anaerobic conditions. The mechanistic insights obtained in the present study could be used in the production of tailor-made protein aggregates for future food applications, although further investigations on process optimization are required.

## Figures and Tables

**Table 1:** Concentration (μM) of Trp oxidation products formed in apo-α-LA (0.705 mM) illuminated for 24 h with added Trp (2.8 mM) under aerobic and anaerobic conditions. Free Trp was separated from α-LA by spin filtration, and each of the fractions was analyzed by UHPLC with UV and fluorescence detection. The α-LA samples were subjected to acidic hydrolysis prior to the UHPLC analysis. Results are presented as mean values ± standard deviations obtained from three independent experiments. LOD; limit of detection.

Oxidation Product	Apo-α-LA Separated from Trp	Apo-α-LA Separated from Trp	Free Trp Separated from Apo-α-LA	Free Trp Separated from Apo-α-LA	LOD (µM)
	Aerobic	Anaerobic	Aerobic	Anaerobic	
3-OH-Kyn	56 ± 7	56 ± 4	nd	nd	2.2
NFK	nd	nd	nd	nd	1.2
Kyn	nd	nd	58 ± 2	nd	1.0
5-OH-Trp	nd	nd	11 ± 6	nd	5.2
Tryptamine	nd	nd	2 ± 0	3 ± 1	0.1

## Data Availability

Not applicable.

## References

[1] Díaz O., Pereira C.D., Cobos A. (2004). Functional properties of ovine whey protein concentrates produced by membrane technology after clarification of cheese manufacture by-products. Food Hydrocoll..

[2] Ryan K.N., Foegeding E.A. (2015). Formation of soluble whey protein aggregates and their stability in beverages. Food Hydrocoll..

[3] Bryant C.M., McClements D.J. (1998). Molecular basis of protein functionality with special consideration of cold-set gels derived from heat- denatured whey. Trends Food Sci. Technol..

[4] De Wit J.N. (1998). Nutritional and Functional Characteristics of Whey Proteins in Food Products. J. Dairy Sci..

[5] White S.S., Fox K.M., Jervis S.M., Drake M.A. (2013). Influence of heating and acidification on the flavor of whey protein isolate. J. Dairy Sci..

[6] Jansson T., Nielsen S.B., Petersen M.A., Lund M.N. (2020). Temperature-dependency of unwanted aroma formation in reconstituted whey protein isolate solutions. Int. Dairy J..

[7] Zhao Z., Engholm-Keller K., Poojary M.M., Boelt S.G., Rogowska-Wrzesinska A., Skibsted L.H., Davies M.J., Lund M.N. (2020). Generation of Aggregates of α-Lactalbumin by UV-B Light Exposure. J. Agric. Food Chem..

[8] Vanaman T.C., Brew K.H.R. (1970). The disulfide bonds of bovine alpha-lactalbumin. J. Biol. Chem..

[9] Gammelgaard S.K., Petersen S.B., Haselmann K.F., Nielsen P.K. (2020). Direct Ultraviolet Laser-Induced Reduction of Disulfide Bonds in Insulin and Vasopressin. ACS Omega.

[10] Asquith R.S., Hirst L. (1969). The photochemical degradation of cystine in aqueous solution in the presence of air. BBA Gen. Subj..

[11] Vanhooren A., Devreese B., Vanhee K., Van Beeumen J., Hanssens I. (2002). Photoexcitation of tryptophan groups induces reduction of two disulfide bonds in goat α-lactalbumin. Biochemistry.

[12] Pattison D.I., Rahmanto A.S., Davies M.J. (2012). Photo-oxidation of proteins. Photochem. Photobiol. Sci..

[13] Correia M., Neves-Petersen M.T., Parracino A., Di Gennaro A.K., Petersen S.B. (2012). Photophysics, photochemistry and energetics of UV light induced disulphide bridge disruption in apo-α-lactalbumin. J. Fluoresc..

[14] Bent D.V., Hayon E. (1975). Excited state chemistry of aromatic amino acids and related peptides. III. Tryptophan. J. Am. Chem. Soc..

[15] Bent D.V., Hayon E. (1975). Excited state chemistry of aromatic amino acids and related peptides. I. Tyrosine. J. Am. Chem. Soc..

[16] Zhao Z., Poojary M.M., Skibsted L.H., Lund M.N., Lund M.N. (2020). Cleavage of Disulfide Bonds in Cystine by UV-B Illumination Mediated by Tryptophan or Tyrosine as Photosensitizers. J. Agric. Food Chem..

[17] Nielsen L.R., Nielsen S.B., Zhao Z., Olsen K., Nielsen J.H., Lund M.N. (2018). Control of α-Lactalbumin Aggregation by Modulation of Temperature and Concentration of Calcium and Cysteine. J. Agric. Food Chem..

[18] Zainudin M.A.M., Poojary M.M., Jongberg S., Lund M.N. (2019). Light exposure accelerates oxidative protein polymerization in beef stored in high oxygen atmosphere. Food Chem..

[19] Gomyo T., Fujimaki M. (1970). Studies on Changes of Protein by Dye Sensitized Photooxidation. Agric. Biol. Chem..

[20] Haywood J., Mozziconacci O., Allegre K.M., Kerwin B.A., Schöneich C. (2013). Light-induced conversion of trp to gly and gly hydroperoxide in IgG1. Mol. Pharm..

[21] Neves-Petersen M.T., Klitgaard S., Pascher T., Skovsen E., Polivka T., Yartsev A., Sundström V., Petersen S.B. (2009). Flash photolysis of cutinase: Identification and decay kinetics of transient intermediates formed upon UV excitation of aromatic residues. Biophys. J..

[22] Dose K. (1968). The photolysis of cystine in the presence of aromatic amino acids. Photochem. Photobiol..

[23] Creed D. (1984). The photophysics and photochemistry of the near-uv absorbing amino acids-III. Cystine and its simple derivatives. Photochem. Photobiol..

[24] Kristo E., Hazizaj A., Corredig M. (2012). Structural changes imposed on whey proteins by UV irradiation in a continuous UV light reactor. J. Agric. Food Chem..

[25] Catalfo A., Bracchitta G., De Guidi G. (2009). Role of aromatic amino acid tryptophan UVA-photoproducts in the determination of drug photosensitization mechanism: A comparison between methylene blue and naproxen. Photochem. Photobiol. Sci..

[26] Hellwig M. (2019). The Chemistry of Protein Oxidation in Food. Angew. Chem. Int. Ed..

[27] Dalsgaard T.K., Otzen D., Nielsen J.H., Larsen L.B. (2007). Changes in structures of milk proteins upon photo-oxidation. J. Agric. Food Chem..

